# Medicine

**Published:** 1897-06

**Authors:** George Parker


### Progress of the Medical Sciences.

### MEDICINE.

The causation of **cirrhosis of the liver**, its course and diagnosis, have been the subject of several interesting recent discussions, and certain new facts seem to have been arrived at by a closer investigation of the phenomena. In alcoholic cirrhosis, as Sir Dyce Duckworth[1] remarks, the malady is far from being a local one, for simultaneous changes affect almost every tissue of the body, and with the appearance of cirrhosis a gradual cachexia supervenes. If, indeed, the liver is not the weak point of the

[1] *Practitioner*, 1897, lviii. 235.

individual, failure may result in other organs long before there is time for the liver to be affected. Thus, in young women the nervous system is more vulnerable, and neuritis is a more frequent result of alcoholic poisoning in them. Besides the hypertrophic liver of non-alcoholic origin where there is much jaundice, Duckworth recognises two alcoholic forms of cirrhosis, the atrophic and hypertrophic, either of which may be part of the general systemic change produced by the habit.

A curious thing, however, is that **cirrhosis of the kidney,** though common enough by itself in alcoholic subjects, according to some authorities, is **not often associated with that of the liver.** Pitt[1] found that in eighty-nine cases of cirrhosis the kidneys weighed over ten ounces in thirty-eight of the number, and but eighteen showed granular disease; while Dickinson, in two series of 149 persons each, found that the number of granular kidneys was the same in liquor dealers and in apparently temperate persons. If we discredit the latter argument, still the comparatively rare coincidence of the two diseases, cirrhosis of the liver and that of the kidney, is remarkable, whether this arises from the small amount of alcohol excreted by the kidney, or because the organ which happens to be functionally stronger in an individual deals with the poison and shields the other, is uncertain. When the two are affected together, fatal coma may develop, as Levi has shown.

The question of the **mode of causation** then arises: Does alcohol act directly on the tissues of the liver, or does it inhibit some protective mechanism, so that they are exposed to the action of a toxin developed elsewhere in the body? Now, leaving on one side the hypertrophic forms of cirrhosis, both those of alcoholic and other origins, there are undoubtedly many cases of cirrhosis, indistinguishable from the true alcoholic ones, in which the disease is clearly due to a different irritant. It seems clear that certain cases in children, even in the newly born, are to be found where alcohol has never been administered; and, again, phosphorus and the syphilitic virus are equally able to produce the small cirrhosed liver under favourable conditions, without the presence of alcohol. In the kidney, again, gout, lead, and many other poisons, acting for a considerable period, are more frequent causes than alcohol. Finally, in animals, though cirrhosis is not unknown, especially in cats, it seems extremely difficult to bring it on, according to several observers, by the excessive and long-continued administration of alcohol. But, on the other hand, it is easy to induce the disease by infecting animals with pyogenic microbes, or their toxins. Charrin, Roger, and lastly Krawkow have shown this by numerous experiments. Krawkow[2] injected the microbes into the intestinal canal, and also noted similar results where chronic infection by septic

[1] *A System of Medicine*, edited by T. C. Allbutt, 1897, vol. ii., p. 861.
[2] *Arch. de Méd. expér. et d'Anat. path.*, 1896, viii. 106; abstract in *Brit. M. J.*, 1896, ii. Epitome, p. 80.

organisms had occurred, while amyloid degeneration followed the injection of these poisons into the muscles. These considerations lend some weight to the theory that the cirrhosis of drunkards is due to a secondary infection. By the constant abuse of alcohol, the stomach and intestines are in a chronic state of congestion, if not of ulceration, and no longer exert their protective and selective power, so that various toxins are absorbed, which attack the liver. If, too, it was a direct physical action of alcohol, we should hardly expect to find the absolute immunity from cirrhosis which is occasionally seen in some very hard drinkers, though we grant the varying degree of resistance in different individuals. Thus, Formad found it only in six cases out of 250 necropsies on confirmed drunkards. It may be said, however, that if alcohol is broken up in the body, the cirrhosis is produced by substances formed from it. Thus, Boix produced cirrhosis by the administration of lactic, acetic, butyric, and valerianic acids. But even if any of these are formed by the disintegration of alcohol, it is clear that they are often produced without it in ordinary dyspepsia. Douglas Stanley[1] gives an interesting account of these investigations. He discusses the effects of gastric disorders in producing diseases of the liver, and refers to the occurrence of hypertrophy in dilatation of the stomach, and in children affected with gastro-intestinal disease, as well as in some tubercular patients. Hayem is also quoted as holding that atrophic cirrhosis is coincident with chronic gastritis where hyperpepsia exists.

On the whole, there is a good deal of evidence that **atrophic cirrhosis can be produced by other causes than alcohol,** and that it even follows some acute fevers, as well as microbic infections and digestive disturbances, while in the common alcoholic form it is doubtful whether the change is due to the direct action of the drug on the epithelial cells or on the connective tissues, or, finally, to the results of the chronic dyspepsia set up by it. Stanley notices that animals in whom cirrhosis was set up by organic acids lived actually longer if alcohol was given at the same time.

Foxwell, Fenton, Rolleston, and Kelynack have recently given numerous statistics on the size, weight, and other **pathological details of the cirrhosed liver:** Kelynack[2] takes 121 cases, mostly alcoholic, though the distinction is not easy to make in hospital patients; while Rolleston and Fenton[3] give an analysis of 114. The forms of biliary and infantile cirrhosis are excluded from both series. Kelynack found that nearly fifty per cent. die directly from the liver disease, and over twelve per cent. from tuberculosis. Cirrhosis appeared most frequent in males, though fatal at an earlier date in women. The weight of the liver was on the average increased, while the size was diminished in two-thirds of the cases *post mortem*, though possibly many of these were

1 *Birmingh. M. Rev.*, 1897, xli. 164. 2 *Ibid.*, 86.
3 *Ibid.*, 1896, xl. 193.

enlarged during life. Renal cirrhosis occurred in only eighteen per cent. Fenton and Rolleston confirm his views as to the frequency of tubercular disease. They hold that the livers in cirrhosis are, on the average, much increased in weight, especially the alcoholic ones, whether these are due to beer or spirits; but where granular kidneys exist in addition, there is very little increase. In young women a large liver, with head symptoms and little ascites, is often found, while in older women ascites and a smaller liver are more frequent. Now, as Kerr points out,[1] most observers are agreed that both enlargement and diminution in weight and size may occur, but (1) some hold there are two distinct varieties, (2) others that a large liver may contract in later stages into a small one, and (3) some think that fatty degeneration is a more important result of alcoholism than cirrhosis. He himself accepts the last view. Do our statistics afford any answers to these questions? According to Rolleston it seems that the kind of alcohol used has no effect on the weight, and that the heavy liver is found more often earlier in life, while as age advances the weight diminishes. If we suppose that the alcoholic habit commenced at the same average age in both types, then the heavy liver represents a more rapidly fatal form than the light one, the patients dying about three years earlier. But it is possible that the heavy livers of arrested or latent cirrhosis found in persons killed by accidents may be due to regeneration or new growth of liver tissue. Finally, heavy and light livers do not differ in the amount of fatty changes; for these occur equally in both classes, and apparently quite as often from spirit drinking as from beer. Though Kelynack, who alone estimates the size as distinct from the weight, finds the size most often decreased *post mortem*, he confesses this is no guide to the size during life. A. Foxwell, in his papers[2] on "The Enlarged Cirrhotic Liver," shows from a large number of cases that an increase of size is the rule in life. After a drinking bout the liver enlarges as much as two or three inches in the twenty-four hours, which may afterwards disappear; and in the great majority of cases he finds that the liver of an alcoholic can be easily felt below the costal margin in the nipple line, even long after the habit has been given up. In sixty-seven cases the average width below the edge of the ribs was $1\frac{6}{11}$ inches, and in twenty-one others it was three inches. He confirms, too, Rolleston's statement that the increase of size in large livers is not due to fatty infiltration, which is contrary to the common doctrine of the text-books.

In view of the little value of most drugs in the **treatment of ascites** due to cirrhosis, the recommendation of urea as a diuretic in cases where the kidneys are intact is worth recording. Friedreich[3] and Klemperer have noticed an enormous increase of the urinary excretion after its administration. It may be

[1] *Med. Chron.*, 1895–96, N.S. iv. 225, 310. [2] *Birmingh. M. Rev.*, 1896, xxxix. 129, 215.
[3] *Orvosi hetil.*, 1896, xl. 41; see *Birmingh. M. Rev.*, 1896, xl. 308.

given in milk or as a powder with carbonate of soda and cinnamon. As much as twenty grammes in a day have thus been taken without ill effects, and it may be continued in daily doses of somewhat smaller amounts.

Failure of the **stomach** to pass on the food, or **dilatation**, is so easily confused with other disorders that Pepper and Stengel's[1] discussion of its diagnosis will be welcome to many. In the first place, they point out that we ought to include under this heading those cases where the stomach fails to empty itself between meals, though there is no change in size of the organ. On the other hand, the variations in size and position of the stomach, while the motor activity is unimpaired, are so common that they are often mistaken for true dilatation. Thus another authority, Bial,[2] finds that dislocation of the stomach from its normal position is extremely frequent, with few or no gastric symptoms, in emphysema and deformed chests; while tight lacing, repeated pregnancies, a bulky diet, and other causes may enlarge the stomach or alter its situation to a marked degree. Pepper notices that the capacity of apparently normal stomachs may vary between 500 and 2,600 cub. centimetres, and possibly more. He would take account, however, both of the size and motor power, for there may be a compensatory increase of the latter in some enlarged stomachs for a time, which may break down eventually, just as we see in similar affections of the heart. Furthermore, he finds that the size is best determined by auscultatory percussion, the bell of the stethoscope being held by the patient over the stomach-area, and percussion being made in lines radiating from that point. A stomach-tube is then passed, and air injected into the organ by an ordinary syringe, so that it is distended and that the size may be clearly mapped out. Finally, to determine the motor failure, a test meal of meat soup and bread is given and the contents withdrawn more than seven hours afterwards. If there is dilatation part of the meal can be found in the organ for a long period beyond the seven hours, and leads to sour and fœtid vomiting, an evening meal often remaining in the stomach till the morning. In this way we can diagnose the disease from megalo-gastria, gastrectasis and gastroptosis, as well as from chronic gastric catarrh. Among the great variety of means proposed in late years these seem to be the simplest and best adapted to the purpose. Inspection, simple percussion, and the splashing sound may give us some idea of the conditions, but are very liable to error, while trans-illumination even if trustworthy requires skill and complicated instruments.

We seem to have learned nothing as to the poisons which

[1] *Am. J. M. Sc.*, 1897, cxiii. 34.

[2] *Berl. klin. Wchnschr.*, 1896, xxxiii. 1107; abstract in *Brit. M. J.*, 1897, i. Epitome, p. 17.

cause **uræmia**, except that they are many, and some are proteids. Urea, carbonate of ammonia, potash salts, and ptomaines have each been invoked in turn. Nor are we certain how the real poisons act; how, for instance, the localised symptoms and more or less transient paralyses are produced. The theory that œdema of the brain causes them is unsupported by facts, and if it exists, œdema certainly often exists nowhere else in the body. Vascular spasm and cerebral anæmia are contradicted by Leonard Hill's researches on the cerebral circulation. He showed that nothing like vaso-motor action occurs in that area. In place of contraction and relaxation of the vessels, the blood-supply seems to be regulated by changes in the splanchnic and other areas. Bradford[1] would refer the convulsions and paralyses to a direct action of the toxins on the nerve centres, some of which happen to be more irritable than others. The very diagnosis of uræmic convulsions from those caused by meningitis is at times impossible. Every focal and general sign, says A. R. Edwards,[2] known to occur in the latter has been observed also in the former. Albumen and casts may be absent, and even the amount of urea and urine may be normal at the time.

In **treatment** one of the most promising methods seems to be that of the injection of artificial serum combined with bleeding, or as Barré terms it[3] "disintoxication of the blood." Simple injection of serum into the tissues or rectum is not satisfactory when the kidneys are imperfect; but in severe and almost hopeless attacks of uræmia, bleeding followed by saline injections has been found not only to restore the patient to consciousness, but even sometimes to put an end to uræmic manifestations altogether. Thus Rendu successfully treated a case of uræmic aphasia by bleeding, and the daily injection of forty centigrammes of artificial serum.[4] Foxwell reported two cases in which rectal injections of saline fluid were used alone.[5] One of the patients quickly recovered, but in the other the advantages were not clear. Richardière[6] had a comatose patient with râles all over the chest and Cheyne-Stokes respiration, whom he bled to the extent of ten ounces, and then injected with twenty-six ounces of Hayem's serum. The patient regained consciousness, and when a relapse occurred two more injections were given with entire disappearance of the uræmia. The temperature after these injections rises a degree or two, the pulse is slowed, diuresis and a useful diarrhœa often follow. If we are to get the full advantage of this method of treatment we should not omit thorough purgation and disinfection of the intestinal tract, together with a milk diet. The latter is of value not only from its action on the kidneys, but also from the

[1] *Practitioner*, 1896, lvii. 293. [2] *Am. J. M. Sc.*, 1896, cxii. 191.
[3] See *Med. Press & Circ.*, 1896, [cxii.] 600.
[4] *Med. Week*, 1896, iv. 164. [5] *Birmingh. M. Rev.*, 1895, xxxviii. 257.
[6] *Union méd.*, 1896, 4e sér. ii. 578.

reduction of the supply of toxic matters, and the extraordinary diminution of fermentative organisms in the intestines which its use brings about. Saundby speaks of benzoate of soda as useful, but whether this is of value apart from cases of ascending infection from the bladder is uncertain. Attempts to influence the skin may also be made by hot packs and hot-air baths, but it is an open question how far we are justified in employing pilocarpin from its well-known risks. To obviate these, it has been recommended to give digitalis previously, or to use the pilocarpin as an ointment of the strength of a grain to the ounce. In some attacks it has considerable value.

An interesting attempt to apply **surgical methods** to those cases where uræmia is threatened by congestive anuria has been made by Reginald Harrison[1] and others. An incision is made over one kidney with antiseptic precautions, and the organ punctured or the capsule incised. Remarkable restoration of the excretory function has followed in some cases, and the good effects produced by the relief of tension are transmitted to the other kidney by a kind of reflex action. The operation, which was suggested by the recoveries following unsuccessful explorations for supposed calculi, is also recommended by Harrison from the similarity of the conditions to those in the eye and the testicle. In both these organs acute inflammation leads to rapid destruction of the structure unless the tension is relieved by incision. E. Hurry Fenwick[2] lays stress on the tenderness with which these congested kidneys must be handled, for the tissues are so engorged as to break up under very slight compression. The method seems applicable to those cases of acute nephritis where the inflammation has become so severe that more or less complete suppression has occurred; and possibly to more chronic ones where no progress seems to be made, and the kidney remains tender when touched.

**Tubercular kidney disease** so frequently develops as a purely local malady that its detection and treatment are highly important. Hæmaturia, frequent micturition, increased mucus in the urine, and later on pus may appear. Meyer notices the congested condition of the mouth of one ureter as seen by the cystoscope.[3] There may also be often pyrexia as well as severe pain, tenderness, and, after a time, a tumour. The urine remains acid even to an advanced stage of the disease; albumen may occur in excess of that due to the pus present and may be estimated separately. For this Reinecke[4] counts the pus corpuscles under the hæmocytometer, estimating that 100,000 pus cells in a cub. cen. produce one per cent. of albumen. The detection of

[1] *Brit. M. J.*, 1896, ii. 1126. [2] *Ibid.*, 1352.
[3] *Med. News*, 1896, lxviii. 253; abstract in *Med. Chron.*, 1896, N.S. v. 278.
[4] *Berl. klin. Wchnschr.*, 1895, xxxii. 1069; abstract in *Internat. M. Mag.*, 1897, v. 120.

tubercle bacilli is not only difficult from their fewness, but they are apt to be confused with the common smegma bacillus; which stains in an almost exactly similar manner. In fact, it seems quite impossible to distinguish them except by washing the slides (after staining) for ten minutes in alcohol, which decolourises the smegma microbe, while the tubercular one retains its colour. As to the origin of the disease, Hamill[1] brings forward a good deal of evidence that it is carried down from the blood rather than upwards from the bladder in nearly all cases, that the bladder is unaffected in half the number; and, finally, that the disease is most frequently unilateral. Hence he advocates early surgical interference before the lower urinary passages are affected. R. Harrison[2] notices the origin of urinary tubercle in some cases by extension from the peritoneum, and regards surgical aid as not often likely to be of use. Tuffier,[3] while he recognises the existence of descending unilateral localised disease, thinks operation should be restricted to cases where the patient is sinking from pain, hemorrhage, septic or tubercular intoxication, or where general infection is feared. It seems certain that though many of these patients perish miserably others recover completely without operation. As to medical treatment, Harrison recommends small doses of corrosive sublimate in the early stages, and morphia for the pain. Cod liver oil, creasote, calcium chloride, and the ordinary means used in the treatment of phthisis must be employed. Urotropin, a formalin compound, gives promise of some utility from its remarkable action on the urinary tract, and possibly one of the improved forms of tuberculin may be found of value.

George Parker.

### SURGERY.

1 *Internat. M. Mag.*, 1896, iv. 881.

2 *Twentieth Century Practice of Medicine*, edited by T. L. Stedman, vol. i., 1895, p. 154.

3 *Med. Week*, 1897, v. 33.

